# ﻿Two new species of *Samsoniella* (Cordycipitaceae, Hypocreales) from the Mayao River Valley, Guizhou, China

**DOI:** 10.3897/mycokeys.99.109961

**Published:** 2023-09-15

**Authors:** Wan-Hao Chen, Jian-Dong Liang, Xiu-Xiu Ren, Jie-Hong Zhao, Yan-Feng Han

**Affiliations:** 1 Center for Mycomedicine Research, Basic Medical School, Guizhou University of Traditional Chinese Medicine, Guiyang 550025, Guizhou, China Guizhou University of Traditional Chinese Medicine Guiyang China; 2 Institute of Fungus Resources, Department of Ecology, College of Life Sciences, Guizhou University, Guiyang 550025, Guizhou, China Guizhou University Guiyang China

**Keywords:** Entomopathogenic fungi, morphology, phylogenetic analysis, Sordariomycetes, valley

## Abstract

*Samsoniella* species have been often found in the forest habitat and rarely found in special karst eco-environments, such as Tiankeng, valleys and caves. In this research, eleven cordyceps specimens were collected from Mayao River Valley. A known species (*S.haniana*) and two new species (*S.duyunensis* and *S.vallis*) were established and described according to a multilocus phylogenetic analysis and morphological characteristics. Our results provide insight that the richness of *Samsoniella* species in karst eco-environments and further attention should be paid to entomopathogenic fungi in such habitats.

## ﻿Introduction

The genus *Samsoniella* Mongkols., Noisrip., Thanakitp., Spatafora & Luangsa-ard was proposed based on the phylogenetic analysis of *Isaria*-like morphs in Cordycipitaceae and characterised by oval to fusiform conidia and bright red-orange teleomorphic stromata and anamorphic synnemata by Mongkolsamrit et al. (2018). One *Isaria*-like species, *Penicilliumalboaurantium* G. Sm. was transferred to *Samsoniella* and two new species, *S.aurantia* and *S.inthanonensis* were described.

Subsequently, Chen et al. (2020a) reported three new species, *S.coleopterorum* W.H. Chen, Y.F. Han & Z.Q. Liang, *S.hymenopterorum* W.H. Chen, et al. and *S.lepidopterorum* W.H. Chen, et al. from the forestry of Xishui and Rongjiang County, Guizhou Province, China. Those species have mononematous conidiophores rather than synnemata and associated with hymenopteran larvae, coleopteran larvae and lepidopteran pupae, respectively. Wang et al. (2020) described nine new species and a new combination from the forest habitats of Yunnan Province, China. The other thirteen new species were reported by Chen et al. (2021a, 2022), Wang et al. (2022) and Crous et al. (2023). Currently, there are twenty-nine species described in the genus of *Samsoniella*.

Additionally, it has been reported that *Samsoniella* species are found in the forest habitat. However, the other ecological habitats, especially the karst eco-environment which has special niches like Tiankeng, valleys and caves should have insects and entomopathogenic fungi. In this research, eleven cordyceps specimens were collected from Mayao River Valley, Guizhou, China. After detailed multiloci phylogenic analysis and morphological observation, two new species and one known species were identified.

## ﻿Materials and methods

### ﻿Specimen collection and identification

Eleven cordyceps specimens were collected from Mayao River Valley (26°22'8.3748"N, 107°23'16.96"E), Duyun City, Qiannan Buyei and Miao Autonomous Prefecture, Guizhou, on 4 September 2021 and 30 July 2022. The samples were placed in an ice box and brought to the laboratory and preserved in refrigerator at 4 °C before use. The surface of each arthropod body was rinsed with sterile water, followed by sterilisation with 75% ethanol for 3–5 s and rinsing again three times with sterilised water. After drying on sterilised filter paper, a piece of the synnemata, mycelium or the sclerotia was cut from the specimen and inoculated on agar plates of potato dextrose agar (PDA) or PDA modified by the addition of 1% w/v peptone containing 0.1 g/l streptomycin and 0.05 g/l tetracycline (Chen et al. 2019a). After fungal colonies emerged from the inoculated samples, a piece of mycelium from the colony edge was transferred onto new agar plated and cultured at 25 °C for 14 days under 12 h light/12 h dark conditions (Zou et al. 2010). The specimens and axenic cultures were deposited at the Institute of Fungus Resources, Guizhou University (formally Herbarium of Guizhou Agricultural College; code, GZAC), Guiyang City, Guizhou, China.

Colony morphology was determined on PDA cultures incubated at 25 °C for 14 days and the growth rate, the presence of octahedral crystals and the colony colours (surface and reverse) were observed. To investigate the microscopic characteristics, a little of the mycelia was picked up from the colony and mounted in lactophenol cotton blue or 20% lactate acid solution and the asexual morphological characteristics (e.g., conidiophores, phialides and conidia) were observed and measured under a Leica DM4 B microscope.

### ﻿DNA extraction, polymerase chain reaction amplification and nucleotide sequencing

DNA extraction was carried out using a fungal genomic DNA extraction kit (DP2033, BioTeke Corporation) according to Liang et al. (2011). The extracted DNA was stored at −20 °C. Polymerase chain reaction (PCR) was used to amplify genetic markers using the following primer pairs: ITS4/ITS5 for the internal transcribed spacer (ITS) region (White et al. 1990), LR0R/LR5 for 28s large subunit ribosomal (LSU) (Vilgalys and Hester 1990), CRPB1/RPB1Cr for RNA polymerase II largest subunit (*RPB1*) (Castlebury et al. 2004), fRPB2-5F/fRPB2-7cR for RNA polymerase II second largest subunit (*RPB2*) (Liu et al. 1999) and 983F/2218R for translation elongation factor 1 alpha (*TEF*) (Castlebury et al. 2004). The thermal cycle of PCR amplification for these phylogenetic markers was set up following the procedure described by Chen et al. (2021). PCR products were purified and sequenced at Sangon Biotech (Shanghai) Co. The resulting sequences were submitted to GenBank (Table 1).

**Table 1.** List of strains and GenBank accession numbers of sequences used in this study.

Species	Strain	Host/Substratum	GenBank accession number					Reference
			ITS	LSU	RPB1	RPB2	TEF	
*Akanthomycesaraneosus*	KY11341	Araneae (Spider)	ON502826	ON502832	–	ON525442	ON525443	Chen et al. (2022)
	KY11342	Araneae (Spider)	ON502844	ON502837	–	ON525444	ON525445	Chen et al. (2022)
*Akanthomycesattenuatus*	CBS 402.78	Leaf litter; *Acersaccharum*	AJ292434	AF339565	EF468888	EF468935	EF468782	Sung et al. (2007)
*Akanthomyceslecanii*	CBS 101247	Hemiptera; *Coccusviridis*	JN049836	AF339555	DQ522407	DQ522466	DQ522359	Spatafora et al. (2007)
*Akanthomycestiankengensis*	KY11571	Araneae (Spider)	ON502848	ON502825	–	ON525446	ON525447	Chen et al. (2022)
	KY11572	Araneae (Spider)	ON502821	ON502827	–	ON525448	ON525449	Chen et al. (2022)
*Akanthomycestortricidarum*	BCC72638	Lepidoptera; tortricidae	MT356076	MT356088	MT477997	MT477992	MT478004	Aini et al. (2020)
*Beauveriabassiana*	ARSEF 1564	Lepidoptera; Arctiidae	HQ880761	–	HQ880833	HQ880905	HQ880974	Rehner et al. (2011)
*Beauveriabrongniartii*	ARSEF 617	Coleoptera; Scarabaeidae	HQ880782	–	HQ880854	HQ880926	HQ880991	Rehner et al. (2011)
	BCC 16585	Coleoptera; *Anomalacuprea* (larva)	JN049867	JF415967	JN049885	JF415991	JF416009	Kepler et al. (2012)
*Samsoniellaalboaurantia*	CBS 240.32	Lepidoptera (pupa)	–	JF415979	JN049895	JF415999	JF416019	Kepler et al. (2012)
	CBS 262.58	Soil	–	AB080087	MF416654	MF416448	MF416497	Kepler et al. (2012)
*Samsoniellaalpina*	YFCC 5818	Hepialidae (*Hepialusbaimaensis*)	–	MN576809	MN576869	MN576923	MN576979	Wang et al. (2020)
*Samsoniellaalpina*	YFCC 5831	Hepialidae (*Hepialusbaimaensis*)	–	MN576810	MN576870	MN576924	MN576980	Wang et al. (2020)
*Samsoniellaantleroides*	YFCC 6016	Noctuidae (Larvae)	–	MN576803	MN576863	MN576917	MN576973	Wang et al. (2020)
	YFCC 6113	Noctuidae (Larvae)	–	MN576804	MN576864	MN576918	MN576974	Wang et al. (2020)
*Samsoniellaaurantia*	TBRC 7271	Lepidoptera	–	MF140728	MF140791	MF140818	MF140846	Mongkolsamrit et al. (2018)
	TBRC 7272	Lepidoptera	–	MF140727	–	MF140817	MF140845	Mongkolsamrit et al. (2018)
*Samsoniellacardinalis*	YFCC 5830	Limacodidae (Pupa)	–	MN576788	MN576848	MN576902	MN576958	Wang et al. (2020)
	YFCC 6144	Limacodidae (Pupa)	–	MN576786	MN576846	MN576900	MN576956	Wang et al. (2020)
*Samsoniellacoccinellidicola*	YFCC 8772	Coccinellidae	–	ON621670	ON676502	ON568685	ON676514	Wang et al. (2020)
	YFCC 8773	Coccinellidae	–	ON621671	ON676503	ON568686	ON676515	Wang et al. (2020)
*Samsoniellacoleopterorum*	A19501	Curculionidae (Snout beetle)	MT626376	–	MT642600	MN101585	MN101586	Chen et al. (2020)
*Samsoniellacristata*	YFCC 6021	Saturniidae (Pupa)	–	MN576791	MN576851	MN576905	MN576961	Wang et al. (2020)
*Samsoniellacristata*	YFCC 6023	Saturniidae (Pupa)	–	MN576792	–	MN576906	MN576962	Wang et al. (2020)
***Samsonielladuyunensis***	**DY09161**	Formicidae (Ant)	**OQ379241**	**OQ363112**	**OR296698**	**OQ397660**	**OQ398145**	This study
	**DY09162**	Formicidae (Ant)	**OQ379242**	**OQ363114**	–	–	**OQ398146**	This study
	**DY07501**	Lepidoptera (Pupa)	**OR263188**	**OR263307**	**OR282773**	**OR282776**	**OR282780**	This study
	**DY07502**	Lepidoptera (Pupa)	**OR263189**	**OR263427**	–	**OR282777**	**OR282781**	This study
*Samsoniellaerucae*	KY11121	Lepidoptera (Caterpillar)	ON502828	ON502835	–	ON525424	ON525425	Chen et al. (2022)
*Samsoniellaerucae*	KY11122	Lepidoptera (Caterpillar)	ON502847	ON502822	–	ON525426	ON525427	Chen et al. (2022)
*Samsoniellafarinospora*	YFCC 8774	Araneae (Spider)	–	ON621672	ON676504	ON568687	ON676516	Wang et al. (2022)
	YFCC 9051	Lepidoptera: *Hepialus*	–	ON621673	ON676505	ON568688	ON676517	Wang et al. (2022)
*Samsoniellaformicae*	KY11041	Formicidae (Ant)	ON502852	–	–	ON525420	ON525421	Chen et al. (2022)
	KY11042	Formicidae (Ant)	ON502842	–	–	ON525422	ON525423	Chen et al. (2022)
*Samsoniellaguizhouensis*	KY11161	Lepidoptera (Pupa)	ON502823	ON502830	–	ON525428	ON525429	Chen et al. (2022)
	KY11162	Lepidoptera (Pupa)	ON502845	ON502846	–	ON525430	ON525431	Chen et al. (2022)
*Samsoniellahaniana*	YFCC 8769	Lepidoptera (Pupa)	–	ON621674	ON676506	ON568689	ON676518	Wang et al. (2022)
	YFCC 8770	Lepidoptera (Pupa)	–	ON621675	ON676507	ON568690	ON676519	Wang et al. (2022)
	YFCC 8771	Lepidoptera (Pupa)	–	ON621676	ON676508	ON568691	ON676520	Wang et al. (2022)
***Samsoniellahaniana***	**DY091031**	Lepidoptera (Pupa)	**OQ359979**	**OQ363133**	–	–	**OQ398149**	This study
	**DY091032**	Lepidoptera (Pupa)	**OQ359978**	**OQ363134**	–	–	**OQ398150**	This study
	**DY091021**	Coccinellidae (ladybug)	**OQ379240**	**OQ363115**	**OR296699**	**OQ397661**	**OQ398147**	This study
	**DY091022**	Coccinellidae (ladybug)	**OQ359881**	**OQ363117**	–	**OQ397662**	**OQ398148**	This study
	**DY091151**	Lepidoptera (Pupa)	**OQ360025**	**OQ363136**	–	–	**OQ398151**	This study
	**DY091152**	Lepidoptera (Pupa)	**OQ360053**	**OQ363137**	–	–	**OQ398152**	This study
*Samsoniellahepiali*	ICMM 82–2	Fungi (*O.sinensis*)	–	MN576794	MN576854	MN576908	MN576964	Wang et al. (2020)
	YFCC 661	Fungi (*O.sinensis*)	–	MN576795	MN576855	MN576909	MN576965	Wang et al. (2020)
*Samsoniellahymenopterorum*	A19521	Vespidae (Bee)	MN128224	–	MT642603	MT642604	MN101588	Chen et al. (2020)
	A19522	Vespidae (Bee)	MN128081	–	–	MN101590	MN101591	Chen et al. (2020)
*Samsoniellainthanonensis*	TBRC 7915	Lepidoptera (Pupa)	MF140761	–	MF140790	MF140815	MF140849	Mongkolsamrit et al. (2018)
	TBRC 7916	Lepidoptera (Pupa)	MF140760	–	–	MF140814	MF140848	Mongkolsamrit et al. (2018)
*Samsoniellakunmingensis*	YHH 16002	Lepidoptera (Pupa)	–	MN576802	MN576862	MN576916	MN576972	Wang et al. (2020)
*Samsoniellalanmaoa*	YFCC 6148	Lepidoptera (Pupa)	–	MN576789	MN576849	MN576903	MN576959	Wang et al. (2020)
*Samsoniellalanmaoa*	YFCC 6193	Lepidoptera (Pupa)	–	MN576790	MN576850	MN576904	MN576960	Wang et al. (2020)
*Samsoniellalepidopterorum*	DL10071	Lepidoptera (Pupa)	MN128076	–	–	MN101593	MN101594	Chen et al. (2020)
	DL10072	Lepidoptera (Pupa)	MN128084	–	–	MT642605	MT642606	Chen et al. (2020)
*Samsoniellaneopupicola*	KY11321	Lepidoptera (Pupa)	ON502843	ON502839	–	ON525432	ON525433	Chen et al. (2022)
	KY11322	Lepidoptera (Pupa)	ON502834	ON502833	–	ON525434	ON525435	Chen et al. (2022)
*Samsoniellapseudogunnii*	GY407201	Lepidoptera (Larvae)	MZ827470	MZ827010	–	MZ855239	MZ855233	Chen et al. (2021)
	GY407202	Lepidoptera (Larvae)	MZ831863	MZ831865	–	MZ855240	MZ855234	Chen et al. (2021)
*Samsoniellapseudotortricidae*	YFCC 9052	Lepidoptera (Pupa)	–	ON621677	ON676509	ON568692	ON676521	Wang et al. (2022)
	YFCC 9053	Lepidoptera (Pupa)	–	ON621678	ON676510	ON568693	ON676522	Wang et al. (2022)
*Samsoniellapupicola*	DY101681	Lepidoptera (Pupa)	MZ827085	MZ827009	–	MZ855237	MZ855231	Chen et al. (2021)
	DY101682	Lepidoptera (Pupa)	MZ827008	MZ827635	–	MZ855238	MZ855232	Chen et al. (2021)
*Samsoniellaramosa*	YFCC 6020	Limacodidae (Pupa)	–	MN576805	MN576865	MN576919	MN576975	Wang et al. (2020)
*Samsoniellasinensis*	YFCC 8766	Lepidoptera (Larvae)	–	ON621679	ON676511	ON568694	ON676523	Wang et al. (2022)
	YFCC 8767	Dermaptera	–	ON621680	ON676512	ON568695	ON676524	Wang et al. (2022)
	YFCC 8768	Dermaptera	–	ON621681	ON676513	ON568696	ON676525	Wang et al. (2022)
*Samsoniellatiankengensis*	KY11741	Lepidoptera (Pupa)	ON502840	ON502838	–	ON525436	ON525437	Chen et al. (2022)
	KY11742	Lepidoptera (Pupa)	ON502849	ON502841	–	ON525438	ON525439	Chen et al. (2022)
*Samsoniellatortricidae*	YFCC 6013	Tortricidae (Pupa)	–	MN576807	MN576867	MN576921	MN576977	Wang et al. (2020)
	YFCC 6131	Tortricidae (Pupa)	–	MN576806	MN576866	MN576920	MN576976	Wang et al. (2020)
***Samsoniellavallis***	**DY07241**	Lepidoptera (Pupa)	**OR263159**	**OR263306**	**OR282772**	**OR282774**	**OR282778**	This study
	**DY07242**	Lepidoptera (Pupa)	**OR263186**	**OR263308**	–	**OR282775**	**OR282779**	This study
	**DY091091**	Lepidoptera (Pupa)	**OR263191**	**OR263428**	–	–	**OR282782**	This study
	**DY091092**	Lepidoptera (Pupa)	**OR263190**	**OR263431**	–	–	**OR282783**	This study
*Samsoniellawinandae*	TBRC 17511	Lepidoptera (Cocoon)	OM491228	OM491231	OM687901	OM687899	OM687896	Crous et al. (2023)
	TBRC 17512	Limacodidae (Pupa)	OM491229	OM491232	OM687902	OM687900	OM687897	Crous et al. (2023)
*Samsoniellayunnanensis*	YFCC 1527	Fungi (*Cordycepscicadae*)	–	MN576812	MN576872	MN576926	MN576982	Wang et al. (2020)
	YFCC 1824	Fungi (*Cordycepscicadae*)	–	MN576813	MN576873	MN576927	MN576983	Wang et al. (2020)

The new strains or species are in bold type.

### ﻿Sequence alignment and phylogenetic analyses

DNASTAR Lasergene (version 6.0) was used to edit DNA sequences in this study. The ITS, LSU, *RPB1*, *RPB2* and *TEF* sequences were downloaded from GenBank, based on Mongkolsamrit et al. (2018), Chen et al. (2020a, 2021a, 2022), Wang et al. (2020, 2022) and Crous et al. (2023) and others selected on the basis of BLASTn searches in GenBank. ITS sequences and other loci were aligned and edited by MAFFT v.7.037b (Katoh and Standley 2013) and MEGA6 (Tamura et al. 2013). Combined sequences of ITS, LSU, *RPB1*, *RPB2* and *TEF* were obtained using SequenceMatrix v.1.7.8 (Vaidya et al. 2011). The model was selected for Bayesian analysis by ModelFinder (Kalyaanamoorthy et al. 2017) in PhyloSuite software (Zhang et al. 2020).

ITS sequences, other loci and the combined loci were analysed using Bayesian inference (BI) and maximum likelihood (ML) methods. For BI, a Markov chain Monte Carlo (MCMC) algorithm was used to generate phylogenetic trees with Bayesian probabilities using MrBayes v.3.2 (Ronquist et al. 2012) for the combined sequence datasets. The Bayesian analysis resulted in 20,001 trees after 10,000,000 generations. The first 4,000 trees, representing the burn-in phase of the analysis, were discarded, while the remaining 16,001 trees were used to calculate posterior probabilities in the majority rule consensus tree. After the analysis was finished, each run was examined using the programme Tracer v.1.5 (Drummond and Rambaut 2007) to determine burn-in and confirm that both runs had converged. ML analyses were constructed with IQ-TREE (Trifinopoulos et al. 2016), using an automatic selection of the model.

### ﻿Genealogical Concordance Phylogenetic Species Recognition (GCPSR) analysis

The Genealogical Concordance Phylogenetic Species Recognition model was applied to analyse the related species. The pairwise homoplasy index (PHI) (Bruen et al. 2006) is a model test based on the fact that multiple gene phylogenies will be concordant between species and discordant due to recombination and mutations within a species. The test was performed in SplitsTree4 (Huson and Bryant 2006) as described by Quaedvlieg et al. (2014) to determine the recombination level within phylogenetically closely-related species using a two-locus concatenated dataset. The new species and their closely-related species were analysed using this model. The relationships between closely-related species were visualised by constructing a split graph, using both the LogDet transformation and splits decomposition options.

## ﻿Result

### ﻿Phylogenetic analyses

In the phylogenetic tree, *Beauveriabassiana* (Bals.-Criv.) Vuill. (ARSEF 1564) and *B.brongniartii* (Sacc.) Petch (ARSEF 617 and BCC 16585) were used as the outgroups. The concatenated sequences (ITS, LSU, *RPB1*, *RPB2* and *TEF*) included 36 species (81 strains) and consisted of 3,579 (ITS, 501; LSU, 775; *RPB1*, 641; *RPB2*, 770; and *TEF*, 892) characters with gaps.

**Figure 1.**
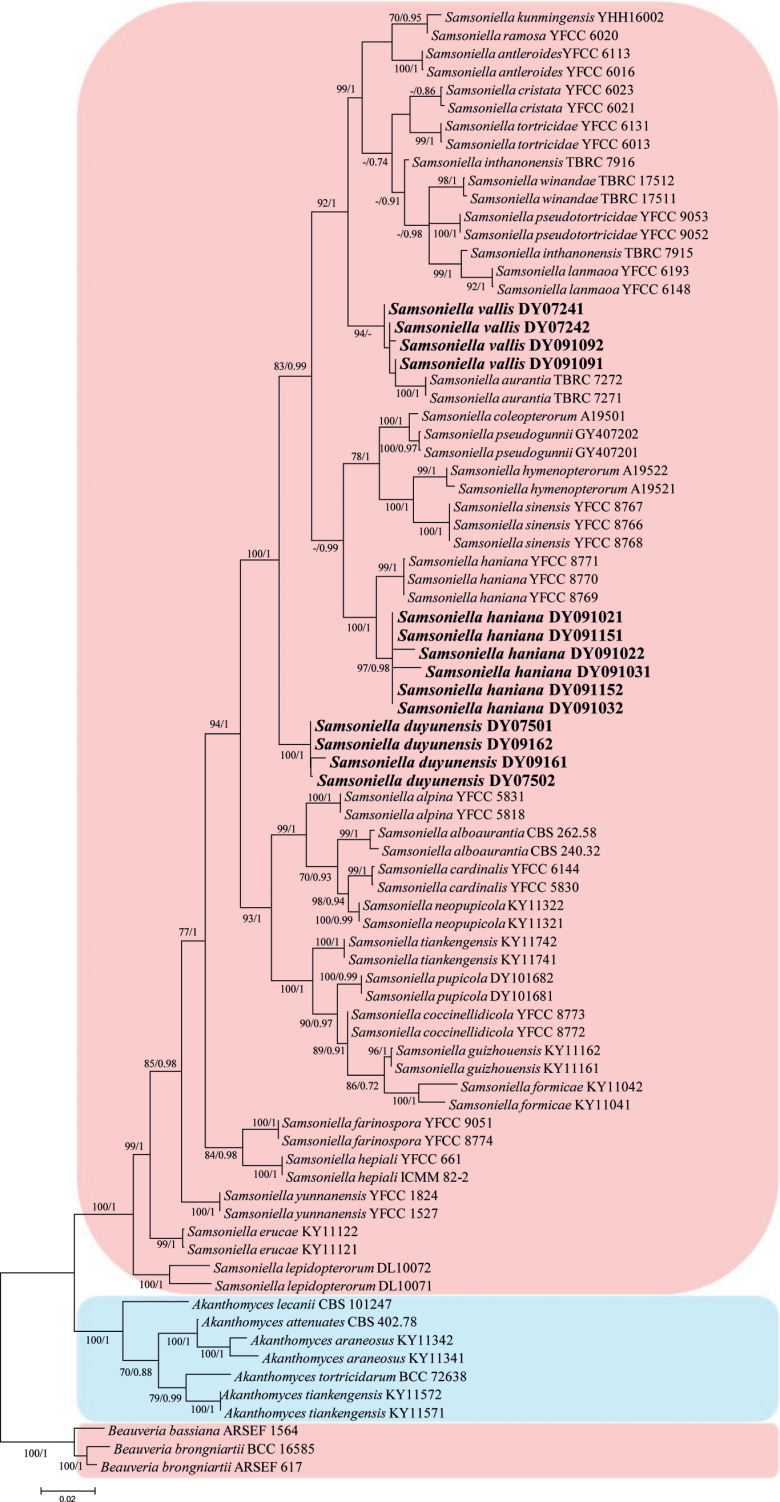
Phylogenetic relationships amongst the new strains and their allies based on multigene dataset (ITS, LSU, *RPB1*, *RPB2* and *TEF*). Statistical support values (≥ 70%/0.70) are shown at the nodes for ML bootstrap support/BI posterior probabilities. The new strains or species are in bold type.

The final value of the highest scoring tree was –15,629.246, which was obtained from the ML analysis of the dataset (ITS+LSU+*RPB1*+*RPB2*+*TEF*). The parameters of the GTR model used to analyse the dataset were estimated, based on the following frequencies: A = 0.235, C = 0.273, G = 0.270, T = 0.222; substitution rates AC = 1.00000, AG = 1.93319, AT = 1.00000, CG = 1.00000, CT = 4.27255 and GT = 1.00000; as well as the gamma distribution shape parameter α = 0.509. The selected models for BI analysis were SYM+G4 (ITS+LSU+*RPB1*+*RPB2*+*TEF*). The phylogenetic trees (Fig. 1), constructed using the ML and BI analyses were largely congruent and strongly supported in most branches. Strains DY091021, DY091022, DY091031, DY091032, DY091151, and DY091152 were clustered into an independent subclade and formed a subclade with *Samsoniellahaniana* Hong Yu bis, Yao Wang & Z.Q. Wang with high statistical support (100% ML /1 PP). Strains DY09161, DY09162, DY07501 and DY07502 were clustered into an independent clade with high statistical support (100% ML/1 PP). Strains DY07241, DY07242, DY091091 and DY091092 were clustered with *S.aurantia* in a clade with high statistical support in ML analysis (94% ML).

**Figure 2.**
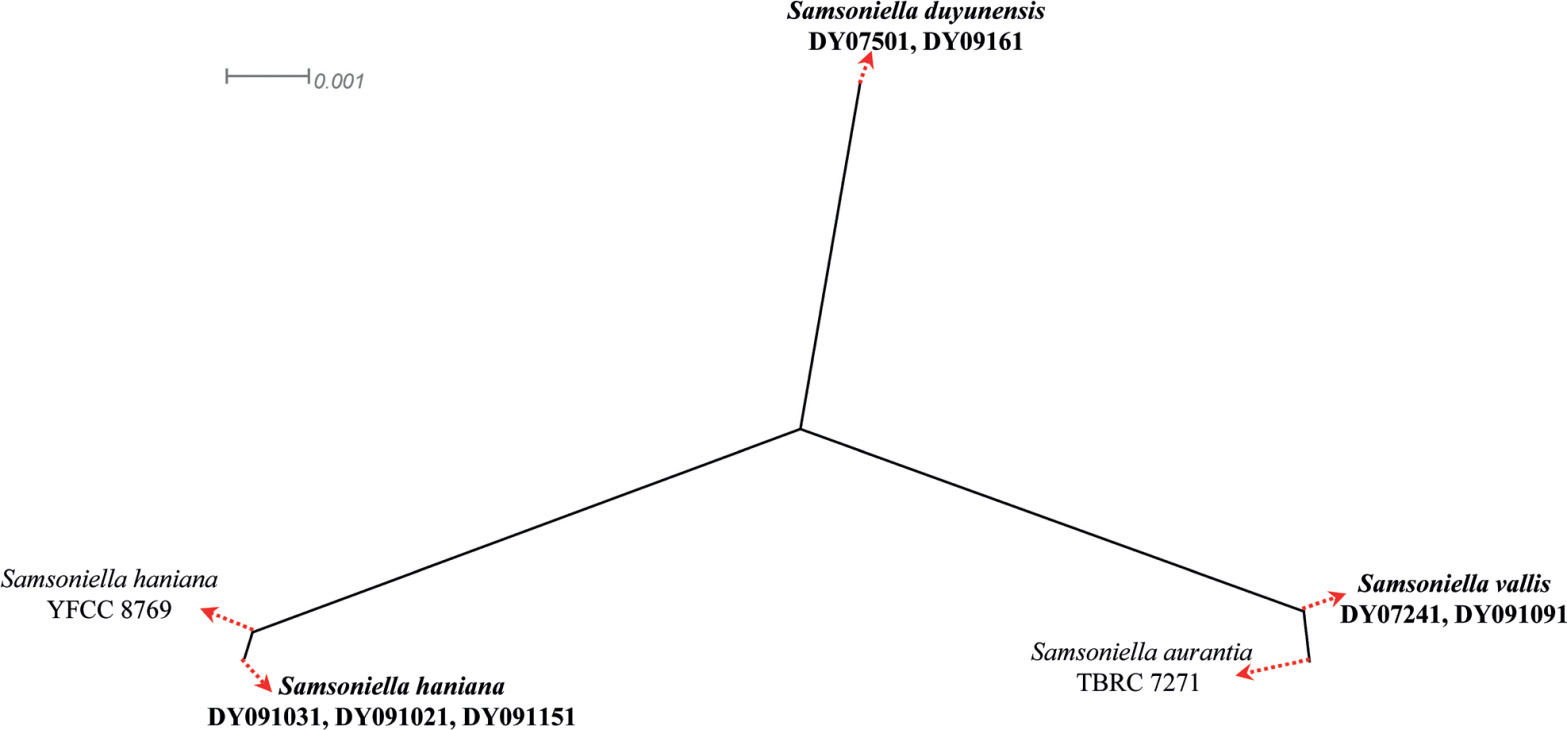
Results of the pairwise homoplasy index (PHI) test of closely-related species using both LogDet transformation and splits decomposition. PHI test results (Փw) < 0.05 indicate significant recombination within the dataset. The new strains or species are in bold type.

A two-locus concatenated dataset (LSU and *TEF*) was used to determine the recombination level within *Samsonielladuyunensis* (DY09161 and DY07501), *Samsoniellavallis* (DY07241 and DY091091), *S.haniana* (YFCC 8769, DY091031, DY091021 and DY091151) and *S.aurantia* (TBRC 7271). Chaiwan et al. (2022) noted that if the PHI is below the 0.05 threshold (Φw < 0.05), it indicates that there is significant recombination in the dataset. This means that related species in a group and recombination levels are not different. If the PHI is above the 0.05 threshold (Φw > 0.05), it indicates that it is not significant, which means the related species in a group level are different. The result of the pairwise homoplasy index (PHI) test of *Samsoniellaaurantia*, *S.duyunensis*, *S.haniana* and *S.vallis* was 1.0 and revealed that the four species were different (Fig. 2).

### ﻿Taxonomy

#### ﻿ Samsoniella duyunensis

Taxon classificationFungiHypocrealesCordycipitaceae

W.H. Chen, Y.F. Han & J.D. Liang sp. nov.

90CA95AD-D8C9-5498-9FEC-6E2375C50DFA

847492

Fig. 3

##### Type.

China, Guizhou, Qiannan Buyei and Miao Autonomous Prefecture, Duyun City, Mayao River Valley (26°22'8.3748"N, 107°23'16.96"E). On an ant (Formicidae), buried in soil, 4 September 2021, Wanhao Chen, GZAC DY0916 (holotype), ex-type living cultures, DY09161.

##### Description.

Synnemata arising from the host, irregularly branched, conidia in abundance at the apex. Colonies on PDA, attaining a diameter of 35–38 mm after 14 days at 25 °C, white, consisting of a basal felt, floccose hyphal overgrowth; reverse yellowish. Hyphae septate, hyaline, pale pink in the middle part, smooth-walled, 0.8–1.4 μm wide. Conidiophores hyaline, smooth-walled, with single phialide or whorls of 2–4 phialides or verticillium-like from hyphae directly, 10.0–21.3 × 1.7–1.9 μm. Phialides cylindrical to ellipsoidal, somewhat inflated base, 5.3–9.1 × 1.3–1.6 μm, tapering to a thin neck. Conidia hyaline, smooth-walled, fusiform to ellipsoidal, 2.1–2.9 × 1.1–1.7 μm, forming divergent and basipetal chains. Sexual state not observed.

**Figure 3.**
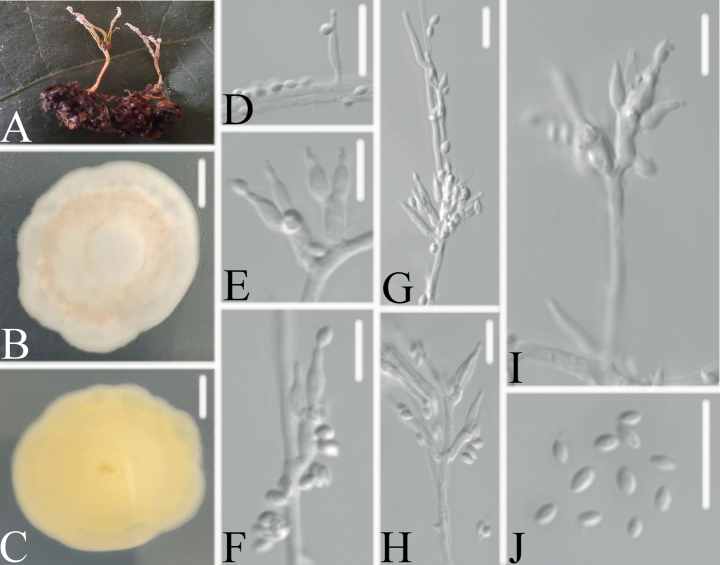
*Samsonielladuyunensis***A** infected ant (Formicidae) **B, C**PDA culture plate showing top (**B**) and reverse (**C**) sides of the colony **D–J** Phialides and conidia. Scale bars: 10 mm (**B, C**); 10 μm (**D–J**).

##### Host.

Ant (Formicidae).

##### Etymology.

Referring to its location in Duyun City.

##### Additional material examined.

China, Guizhou, Qiannan Buyei and Miao Autonomous Prefecture, Duyun City, Mayao River Valley (26°22'8.3748"N, 107°23'16.96"E). On an ant (Formicidae), buried in soil, 4 September 2021, Wanhao Chen, DY09162 (living culture). On a pupa (Lepidoptera) clinging to fallen leaves, 30 July 2022, Wanhao Chen, GZAC DY0750 (specimen), DY07501 and DY07502 (living culture). On an ant (Formicidae) clinging to fallen leaves, 4 September 2021, Wanhao Chen, DY0906 (specimen).

##### Remarks.

*Samsonielladuyunensis* was easily identified as *Samsoniella*, based on the BLASTn result in NCBI and the phylogenetic analysis of the combined datasets (ITS, LSU, *RPB1*, *RPB2* and *TEF*) (Fig. 1) and clustered into an independent clade. Comparing with the typical characteristics of the known species and the keys of *Samsoniella* species (Wang et al. 2022), *S.duyunensis* has a close relationship with *S.coccinellidicola* and *S.sinensis* by absence of sexual state, presence of synnemata and irregularly branched, moderately grow of colony. However, it is distinguished from *S.coccinellidicola* (phialides: 6.0–14.1 × 1.0–2.0 μm; conidia: fusiform or oval, 1.8–3.0 × 1.3–2.0 μm; host, adults of Coccinellidae) by shorter phialides, smaller conidia and its ant host and distinguished from *S.sinensis* (conidia: spherical, elliptical or fusiform; host: larva of Lepidoptera) by fusiform to ellipsoidal conidia and its ant host.

#### ﻿ Samsoniella vallis

Taxon classificationFungiHypocrealesCordycipitaceae

W.H. Chen, Y.F. Han & J.D. Liang sp. nov.

72AC4F73-B0B2-5558-8567-BE546FF52AF1

847493

Fig. 4

##### Type.

China, Guizhou, Qiannan Buyei and Miao Autonomous Prefecture, Duyun City, Mayao River Valley (26°22'8.3748"N, 107°23'16.96"E). On a pupa (Lepidoptera) clinging to fallen leaves, 30 July 2022, Wanhao Chen, GZAC DY0724 (holotype), ex-type living cultures, DY07241.

##### Description.

Synnemata arising from every part of the body of the pupa host. Synnemata erect, usually irregularly branched at the apex, conidia in abundance at the apex. Colonies on PDA, attaining a diameter of 31–37 mm after 14 days at 25 °C, white, consisting of a basal felt, floccose hyphal overgrowth; reverse yellowish. Hyphae septate, hyaline, smooth-walled, 2.1–3.0 μm wide. Conidiophores hyaline, smooth-walled, with single phialide or whorls of 2–4 phialides or verticillium-like from hyphae directly, 11.3–22.1 × 1.3–1.4 μm. Phialides cylindrical to ellipsoidal, somewhat inflated base, 7.2–8.1 × 2.8–3.2 μm, tapering to a thin neck. Conidia hyaline, smooth-walled, fusiform to ellipsoidal, 2.3–3.1 × 1.5–2.1 μm, forming divergent and basipetal chains. Sexual state not observed.

**Figure 4.**
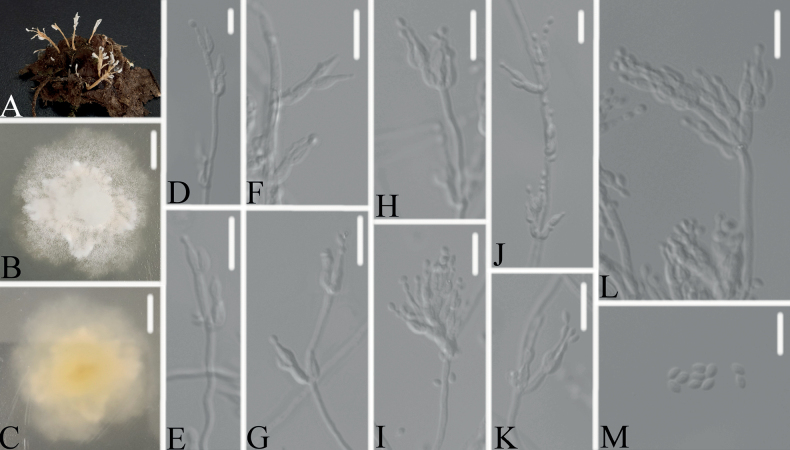
*Samsoniellavallis***A** infected pupa (Lepidoptera) **B, C**PDA culture plate showing top (**B**) and reverse (**C**) sides of the colony **D–M** phialides and conidia. Scale bars: 10 mm (**B, C**); 10 μm (**D–M**).

##### Host.

Pupa (Lepidoptera).

##### Etymology.

Referring to its location in Mayao River Valley.

##### Additional material examined.

China, Guizhou, Qiannan Buyei and Miao Autonomous Prefecture, Duyun City, Mayao River Valley (26°22'8.3748"N, 107°23'16.96"E). On a pupa (Lepidoptera) clinging to fallen leaves, 30 July 2022, Wanhao Chen, DY07242 (living culture); China, Guizhou, Qiannan Buyei and Miao Autonomous Prefecture, Duyun City, Mayao River Valley (26°22'8.3748"N, 107°23'16.96"E). On a pupa (Lepidoptera) clinging to fallen leaves, 4 September 2021, Wanhao Chen, GZAC DY09109 (specimen), DY091091 and DY091092 (living culture). On a pupa (Lepidoptera) clinging to fallen leaves, 4 September 2021, Wanhao Chen, GZAC DY0909 (specimen).

##### Remarks.

*Samsoniellavallis* was easily identified as *Samsoniella*, based on the BLASTn result in NCBI and the phylogenetic analysis of the combined datasets (ITS, LSU, *RPB1*, *RPB2* and *TEF*) (Fig. 1) and clustered with *S.aurantia* in a clade. However, it is distinguished from *S.aurantia* (phialides: 5–13 × 2–3 μm; conidia: fusiform or oval, 2–4 × 1–2 μm) by shorter phialides, smaller fusiform to ellipsoidal conidia. Comparing with the typical characteristics of the known species and the keys of *Samsoniella* species (Wang et al. 2022), *S.vallis* has a close relationship with *S.coccinellidicola* and *S.sinensis* by absence of sexual state, presence of synnemata and irregularly branched, moderate growth of colony. However, it is distinguished from *S.coccinellidicola* (phialides: 6.0–14.1 × 1.0–2.0 μm; conidia: fusiform or oval; host, adults of Coccinellidae) by shorter phialides, fusiform to ellipsoidal conidia and its pupa host and distinguished from *S.sinensis* (phialides: 5.6–9.3 × 1.5–2.1 μm, conidia: spherical, elliptical or fusiform) by fusiform to ellipsoidal conidia and shorter phialides.

#### ﻿ Samsoniella haniana

Taxon classificationFungiHypocrealesCordycipitaceae

Hong Yu bis, Yao Wang & Z.Q. Wang, in Wang, Wang, Dong, Fan, Dao & Yu, Journal of Fungi 8: 20, 2022

B9DF58D0-7DDF-5BEF-9C95-B07240D0727F

Fig. 5

##### Description.

Synnemata arising from every part of the body of the pupa host. Synnemata erect, usually irregularly branched at the apex, *Isaria*-like morph producing a mass of conidia at the branch apex, powdery and floccose. Colonies on PDA, attaining a diameter of 32–35 mm after 14 days at 25 °C, white, consisting of a basal felt, floccose hyphal overgrowth; reverse yellowish. Hyphae septate, hyaline, smooth-walled, 1.3–1.8 μm wide. Conidiophores hyaline, smooth-walled, with single phialide or whorls of 2–8 phialides or verticillium-like from hyphae directly, 16.1–23.9 × 1.7–2.2 μm. Phialides consisting of a cylindrical to ellipsoidal, somewhat inflated base, 5.0–6.9 × 1.8–2.5 μm, tapering to a thin neck. Conidia hyaline, smooth-walled, fusiform to subglobose, 1.7–3.4 × 1.7–2.1 μm, forming divergent and basipetal chains. Sexual state not observed.

**Figure 5.**
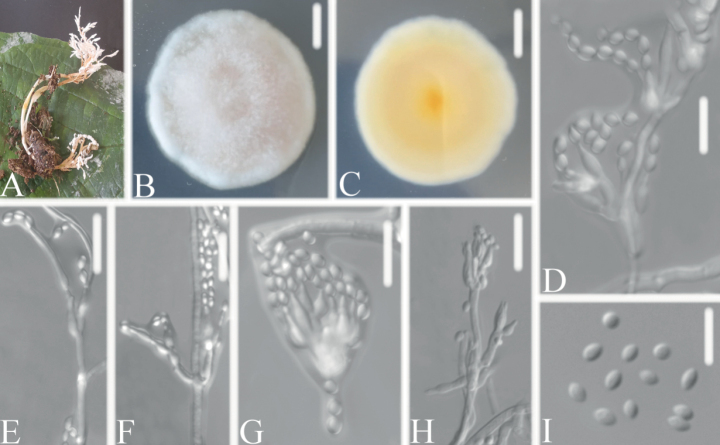
*Samsoniellahaniana***A** infected pupa (Lepidoptera) **B, C**PDA culture plate showing top (**B**) and reverse (**C**) sides of the colony **D–I** phialides and conidia. Scale bars: 10 mm (**B, C**); 10 μm (**D–I**).

##### Host.

Pupa (Lepidoptera).

##### Material examined.

China, Guizhou, Qiannan Buyei and Miao Autonomous Prefecture, Duyun City, Mayao River Valley (26°22'8.3748"N, 107°23'16.96"E). On a pupa (Lepidoptera), buried in soil, 4 September 2021, Wanhao Chen, GZAC DY09103 (specimen), DY091031, DY091032 (living culture); On a ladybug (Coccinellidae); On the moss, 4 September 2021, Wanhao Chen, GZAC DY09102 (specimen), DY091021, DY091022 (living culture); On a caterpillar (Lepidoptera), buried in soil, 4 September 2021, Wanhao Chen, GZAC DY09115 (specimen), DY091151, DY091152 (living culture); On a pupa (Lepidoptera), buried in soil, 4 September 2021, Wanhao Chen, GZAC DY0929, DY09158 (specimen).

##### Remarks.

Strains DY091021, DY091022, DY091031, DY091032, DY091151 and DY091152 were identified as belonging to *Samsoniella*, based on the BLASTn result and the phylogenetic analyses (Fig. 1) and clustered with *S.haniana* in a subclade with high statistical support (100% ML/ 1 PP). The characteristics of those strains were very closely linked with *S.haniana*, which had fusiform or oval conidia (2.3–3.7 × 1.2–2.8 μm), phialide (5.4–12.1 × 1.2–2.9 μm) and a pupa host. Thus, the molecular phylogenetic results and morphologically-based conclusions supported the idea that strains DY091021, DY091022, DY091031, DY091032, DY091151 and DY091152 were *S.haniana*.

## ﻿Discussion

*Samsoniella* species are widely distributed and commonly isolated from soil, insects and spiders or as a fungicolous (Mongkolsamrit et al. 2018; Chen et al. 2020a, 2021a, 2022; Wang et al. 2020, 2022; Crous et al. 2023). Amongst 29 species, *S.alboaurantia*, *S.alpina* H. Yu et al., *S.antleroides* H. Yu et al., *S.aurantia*, *S.cardinalis* H. Yu et al., *S.cristata* H. Yu et al., *S.erucae* W.H. Chen et al., *S.farinospora* Hong Yu bis et al., *S.guizhouensis* W.H. Chen et al., *S.haniana*, *S.hepiali* (Q.T. Chen & R.Q. Dai ex R.Q. Dai, X.M. Li, A.J. Shao, Shu F. Lin, J.L. Lan, Wei H. Chen & C.Y. Shen) H. Yu et al., *S.inthanonensis*, *S.kunmingensis* H. Yu et al., *S.lanmaoa* H. Yu et al., *S.lepidopterorum* W.H. Chen et al., *S.neopupicola* W.H. Chen et al., *S.pseudogunnii* W.H. Chen et al., *S.pseudotortricidae* Hong Yu bis et al., *S.pupicola* W.H. Chen et al., *S.ramosa* H. Yu et al., *S.sinensis* Hong Yu bis et al., *S.tiankengensis* W.H. Chen et al., *S.tortricidae* H. Yu et al., *S.winandae* Mongkols., Noisrip. & Luangsa-ard and *S.yunnanensis* H. Yu et al. were reported as a fungal pathogen of lepidoptera insects. The host of *S.coccinellidicola* Hong Yu bis et al., *S.coleopterorum* W.H. Chen et al., *S.formicae* W.H. Chen et al. and *S.hymenopterorum* W.H. Chen et al. belonged to Coleoptera and Hymenoptera, respectively. In addition, the substrates of *S.alboaurantia*, *S.farinospora* and *S.hepiali* were soil, spider and fungi, respectively. Here, we reported *Samsoniella* species with two different hosts from the valley habitat. More *Samsoniella* species with different hosts or substrates will be reported from diverse habitats.

The taxonomic delimitation of *Samsoniella* was originally based on morphological characteristics and a multi-locus phylogenetic analysis. In the present study, the phylogenetic analysis of a single locus of an individual gene or gene fragment of ITS, LSU, *RPB1*, *RPB2* and *TEF* was tested for the new species (Suppl. materials 1–5) and only the *TEF* could distinguish the new species. However, the new species *S.vallis* could not form an independent clade and clustered with *S.aurantia* as a subclade. A PHI test was added and could solve the taxonomic delimitation of *S.vallis* and *S.aurantia*. Thus, we recommend that the *TEF* locus should be used to distinguish the cryptic *Samsoniella* species and multiple approaches should be used for the further confirmation of a cryptic species.

Generally, species diversity of entomopathogenic fungi were mainly investigated in nature forest and grassland reservations and crop fields (Chen et al. 2019b, 2020b; He 2019; Fan 2020; Zhao et al. 2020, 2021; Zhang et al. 2021). *Samsoniella* species have often been reported from forests, but rarely found in special karst eco-environments, such as Tiankeng, valleys and caves. Chen et al. (2022) reported five new *Samsoniella* species from Monkey-Ear Tiankeng and provided new insights into the richness of species diversity of *Samsoniella* in such special habitat. This research provided further evidence of the richness of *Samsoniella* species in karst eco-environments. The *Samsoniella* species diversity should be extensively investigated in diverse habitats including karst.

## Supplementary Material

XML Treatment for Samsoniella duyunensis

XML Treatment for Samsoniella vallis

XML Treatment for Samsoniella haniana

## ﻿Additional information

### Author ORCIDs

Wan-Hao Chen https://orcid.org/0000-0001-7240-6841

Jian-Dong Liang https://orcid.org/0000-0002-3939-3900

Jie-Hong Zhao https://orcid.org/0000-0003-2972-382X

Yan-Feng Han https://orcid.org/0000-0002-8646-3975

### Data availability

All of the data that support the findings of this study are available in the main text or Supplementary Information.

## Supplementary materials

Supplementary material 1 Phylogenetic relationships among the new strains and their allies based on ITS sequence https://doi.org/10.3897/mycokeys.99.109961.suppl1 Data type tiff Explanation note Statistical support values (≥ 50%/0.50) are shown at the nodes for ML bootstrap support/BI posterior probabilities. The new strains or species are in bold type. https://binary.pensoft.net/file/906245This dataset is made available under the Open Database License (http://opendatacommons.org/licenses/odbl/1.0/). The Open Database License (ODbL) is a license agreement intended to allow users to freely share, modify, and use this Dataset while maintaining this same freedom for others, provided that the original source and author(s) are credited.Wan-Hao Chen, Jian-Dong Liang, Xiu-Xiu Ren, Jie-Hong Zhao, Yan-Feng Han

Supplementary material 2 Phylogenetic relationships among the new strains and their allies based on LSU sequence https://doi.org/10.3897/mycokeys.99.109961.suppl2 Data type tiff Explanation note Statistical support values (≥ 50%/0.50) are shown at the nodes for ML bootstrap support/BI posterior probabilities. The new strains or species are in bold type. https://binary.pensoft.net/file/906246This dataset is made available under the Open Database License (http://opendatacommons.org/licenses/odbl/1.0/). The Open Database License (ODbL) is a license agreement intended to allow users to freely share, modify, and use this Dataset while maintaining this same freedom for others, provided that the original source and author(s) are credited.Wan-Hao Chen, Jian-Dong Liang, Xiu-Xiu Ren, Jie-Hong Zhao, Yan-Feng Han

Supplementary material 3 Phylogenetic relationships among the new strains and their allies based on RPB1 sequence https://doi.org/10.3897/mycokeys.99.109961.suppl3 Data type tiff Explanation note Statistical support values (≥ 50%/0.50) are shown at the nodes for ML bootstrap support/BI posterior probabilities. The new strains or species are in bold type. https://binary.pensoft.net/file/906247This dataset is made available under the Open Database License (http://opendatacommons.org/licenses/odbl/1.0/). The Open Database License (ODbL) is a license agreement intended to allow users to freely share, modify, and use this Dataset while maintaining this same freedom for others, provided that the original source and author(s) are credited.Wan-Hao Chen, Jian-Dong Liang, Xiu-Xiu Ren, Jie-Hong Zhao, Yan-Feng Han

Supplementary material 4 Phylogenetic relationships among the new strains and their allies based on RPB2 sequence https://doi.org/10.3897/mycokeys.99.109961.suppl4 Data type tiff Explanation note Statistical support values (≥ 50%/0.50) are shown at the nodes for ML bootstrap support/BI posterior probabilities. The new strains or species are in bold type. https://binary.pensoft.net/file/906248This dataset is made available under the Open Database License (http://opendatacommons.org/licenses/odbl/1.0/). The Open Database License (ODbL) is a license agreement intended to allow users to freely share, modify, and use this Dataset while maintaining this same freedom for others, provided that the original source and author(s) are credited.Wan-Hao Chen, Jian-Dong Liang, Xiu-Xiu Ren, Jie-Hong Zhao, Yan-Feng Han

Supplementary material 5 Phylogenetic relationships among the new strains and their allies based on TEF sequence https://doi.org/10.3897/mycokeys.99.109961.suppl5 Data type tiff Explanation note Statistical support values (≥ 50%/0.50) are shown at the nodes for ML bootstrap support/BI posterior probabilities. The new strains or species are in bold type. https://binary.pensoft.net/file/906249This dataset is made available under the Open Database License (http://opendatacommons.org/licenses/odbl/1.0/). The Open Database License (ODbL) is a license agreement intended to allow users to freely share, modify, and use this Dataset while maintaining this same freedom for others, provided that the original source and author(s) are credited.Wan-Hao Chen, Jian-Dong Liang, Xiu-Xiu Ren, Jie-Hong Zhao, Yan-Feng Han
